# Understanding Phase-Change Memory Alloys from a Chemical Perspective

**DOI:** 10.1038/srep13698

**Published:** 2015-09-01

**Authors:** A.V. Kolobov, P. Fons, J. Tominaga

**Affiliations:** 1 Nanoelectronics Research Institute, National Institute of Advanced Industrial Science and Technology (AIST), 1-1-1 Higashi, Tsukuba 305-8562, Japan

## Abstract

Phase-change memories (PCM) are associated with reversible ultra-fast low-energy crystal-to-amorphous switching in GeTe-based alloys co-existing with the high stability of the two phases at ambient temperature, a unique property that has been recently explained by the high fragility of the glass-forming liquid phase, where the activation barrier for crystallisation drastically increases as the temperature decreases from the glass-transition to room temperature. At the same time the *atomistic dynamics* of the phase-change process and the associated *changes in the nature of bonding* have remained unknown. In this work we demonstrate that key to this behavior is the formation of transient three-center bonds in the excited state that is enabled due to the presence of lone-pair electrons. Our findings additionally reveal previously ignored fundamental similarities between the mechanisms of reversible photoinduced structural changes in chalcogenide glasses and phase-change alloys and offer new insights into the development of efficient PCM materials.

Non-volatile memory devices are key elements of various electronics and portable systems such as digital cameras, solid state disks, smartphones, computers, e-books, tablets, etc., and their market has been increasing exponentially over the last decade. Even though Flash memory represents today the leading technology, to allow its scalability down to the 16 nm technology node and beyond, new architectures are necessary. Therefore, new emerging non-volatile memory concepts are under investigation and one of the leading candidates is phase-change memory (PCM). PCM has been successfully used in optical memory devices such as DVD-RAM since the 1990s and recently commercial production of electronic non-volatile phase-change random memory (PC-RAM) has been launched by two of the world’s leading memory makers Samsung and Micron.

The basic idea of PCM to utilize the property contrast between the crystalline and amorphous phases (the SET and RESET states) of some materials belongs to S.R. Ovshinsky and dates back to the 1960s^1^. When a material is cooled down slowly, a crystalline phase is formed, when it is cooled down rapidly, an amorphous (glassy) phase is formed. In the phase-change process, a short intense pulse melts the material that is subsequently transformed into the amorphous phase. A longer pulse of lower intensity reverts the material to the crystalline phase. The process is schematically illustrated in Fig. 1 (upper panel). The underlaying phase-change mechanism is generally believed to have a purely thermal origin making use of either Joule heating in electrical memories or the heat released during non-radiative recombination in optical memories (see e.g. a review article^2^). The role of electronic excitation in this process is generally ignored although there is growing experimental and simulational evidence that electronic excitation plays an important role^3^,^4^,^5^,^6^. In order for a material to become commercially interesting, it has to satisfy simultaneously several requirements such as sufficiently large property contrast between the two states and high stability of both phases at operating temperatures alongside with high switching speed in both directions. Less obvious but equally important are the low thermal conductivity of the crystalline phase (to ensure low-energy switching), the relative softness of the material needed to withstand stresses generated at the amorphous-crystalline boundaries allowing for high cyclability, and good scalability. While there are many materials that satisfy some of these requirements, very few satisfy them all. Years of research have singled out GeTe-based PCM alloys. In practical applications, GeTe is usually alloyed with Sb_2_Te_3_ or other additives such as C or N to tune desirable properties, e.g. thermal stability, switching speed or optical contrast (e.g. Ge_2_Sb_2_Te_5_ is used in DVDs but Ge_8_Sb_2_Te_11_ is used in Blu-ray discs). Another class of commercially used materials is the Ag-In-Sb-Te alloys (AIST) used in DVD-RW. In both cases chalcogen atoms are present. In this work we concentrate on GeTe as the simplest composition representing the class of commercially interesting materials for optical memories and the only class used in non-volatile electronic memory devices.

Despite very intense efforts to understand the nature of the phase-change process in GeTe-based alloys and the significant progress achieved, two crucial aspects have remained unaddressed. Essentially all published work discusses the atomic structure of the *two end states* and exclusively from the perspective of the spatial arrangement of atoms in terms of interatomic distances and angles *ignoring the chemical aspects of the interaction* between the atoms. The present work eliminates this shortfall by investigating the dynamics of the structural evolution from the chemical bond perspective.

It should be noted that chalcogenide glasses based on lighter chalcogens, such as sulphur or selenium, also exhibit the ability to change their structure reversibly under exposure to external stimuli such as light. One of the best known examples is reversible photostructural change, where the structure of a chalcogenide glass reversibly changes under light exposure and subsequent annealing close to the glass-transition temperature^7^. Changing the light intensity, wavelength and/or the temperature of exposure can also reverse the process. In contrast to the phase-change alloys, the photostructural transformation takes place entirely within the glassy phase (Fig. 1, lower panel), although under specific conditions photo-crystallisation and photo-amorphysation can also be induced^7^.

Unique features of chalcogenide glasses are the absence of a dark electron spin resonance (ESR) signal, despite a high concentration of defects, and the inability to dope them (i.e. the addition of impurities does not affect the Fermi level position, which remains pinned in the middle of the band gap)^8^. These characteristics were explained by the presence of so called lone-pair (LP) electrons, i.e. pairs of non-bonding valence p-electrons that reside on the same orbital and normally do not participate in the formation of conventional covalent bonds. Kastner introduced the term ‘lone-pair semiconductors’ to refer to this class of materials^9^. The presence of LP-electrons allows the formation of valency alternation pairs (VAPs), which consist of a positively charged three fold-coordinated (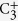) and a negatively charged singly coordinated (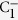) chalcogen atom^10^,^11^,^12^ [whereas the majority of atoms are two-fold coordinated and neutral (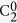)] and exist in concentrations on the order of 10^17^–10^18^ cm^−3^ 8. Because of the electron pairing on the 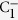 centre, which becomes energetically favourable due to a strong electron phonon coupling, VAPs are said to possess a negative correlation energy (negative-U) and pin the Fermi level of chalcogenides in the middle of the gap^8^.

In the early 1980s Dembovsky proposed that the presence of LP electrons was the underlying reason as to why chalcognides are very good glass formers^13^,^14^. It was proposed that good glass formers possess a concentration of non-bonding LP-electrons *ψ *= (*NVE* − *CN*)/*NVE*, where *NVE* is the number of valence electrons and *CN* is the coordination number, in the range from 0.5 to 0.66 (e.g. 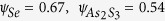
14). He further argued that with appropriate atomic alignment where the LP electrons become aligned with a nearby covalent bond, transient three-centre bonds (TCB) can be generated, providing a natural explanation for the high viscosity of chalcogenide melts, necessary for the glass formation, as well as for the low-energy-barrier bond switching (Figs. 1S and 2S). These processes are discussed in detail in Supplementary Information.

A similar bond-switching process occurs during reversible photostructural changes. For the case of elemental selenium, it was shown that in the photoexcited state the average coordination number increased by ca. 5%, which was interpreted as the formation of transient interchain covalent bonds^15^, with subsequent creation of under-coordinated and over-coordinated Se sites (Cf. Fig. 2S)^16^ in perfect agreement with the idea of VAPs in LP semiconductors^10^. The process can be described as


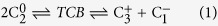


Note that in this process the transient TCBs also play a crucial role. ESR measurements demonstrated that the concentration of photo-induced VAPs can reach 10^20^ cm^−3^ 16,17,18, i.e. several orders of magnitude higher than the concentration of native VAPs. Subsequent computer modelling of this process^19^,^20^ yielded results that were in excellent agreement with the conclusions drawn from the ESR studies. Similar conclusions were reached for binary glasses^17^,^21^. In the binary As_2_S_3_ glass, light exposure additionally resulted in the generation of about 5% of ‘wrong’ As-As bonds^22^. Chalcogenides in the photoexcited state exhibit significantly increased (non-thermal) fluidity^23^,^24^, underscoring the similarity of the processes under thermal and electronic excitation.

In phase-change alloys, chalcogen LP *p*-electrons are used to form dative covalent bonds^25^,^26^ and their role in the phase-change process has been largely neglected. In the crystalline phase, GeTe possesses a distorted rhombohedral structure with the local coordination of 3 (+3) for both species and only Ge-Te bonds exist. In the amorphous phase, the majority of atoms preserve the octahedral bonding geometry^27^,^28^ but with only three covalent Ge-Te bonds, the additional atoms are located at somewhat longer distances^26^,^29^. At the same time, up to 30 to 50% of Ge sites acquire tetrahedral coordination^27^,^28^,^29^,^30^,^31^ concomitant with the formation of homopolar Ge-Ge bonds. An interesting finding was that those Ge sites that were truly tetrahedral contained a Ge first-nearest neighbor^27^,^32^. No Te-Te bonds were detected^33^,^34^. While these studies partially answered the question of how the structure changed as a result of the amorphisation process, the important questions of the bond-switching dynamics remained unanswered.

It should also be noted that in most studies^27^,^28^,^35^, ball-and-stick structure representations are usually used. While this approach was highly productive and helped to identify, for example, the presence of cube-like ABAB (where A = Ge, Sb and B = Te) fragments^28^ in the amorphous phase and the crystallization process was described as the creation of *connected* ‘square rings’^35^, it can also lead to ambiguities in structure determination. Thus, if a bond-length cutoff of 3.2 Å is used, the structure of GeTe appears as distorted rock-salt with all atoms octahedrally coordinated. At the same time, with a cutoff of 3.1 Å, just 0.1 Å smaller, the structure appears as layered with all atoms being three-fold coordinated (Fig. 3S). Obviously, more stringent criteria than just the interatomic distance must be used to determine whether or not the two atoms are covalently bonded. Two most often used approaches to visualise bonding are electron localisation function (ELF)^36^ and charge density difference (CDD)^37^. We have chosen to use the CDD approach because it can show using the same basis both the covalent bonds and lone-pair electrons as demonstrated in Fig. 2. In the example of a selenium chain shown in the upper panel, one can see the CDD clouds located mid-way between atoms that are signatures of covalent bonds alongside with the CDD clouds corresponding to non-bonding *p*-orbitals (lone-pair electrons). In the other example of a hypothetical GeTe cube, which is reminiscent of the ABAB pattern of ref^28^, and can be seen as a building block of the crystalline phase, the bonding angles are very close to 90° and one would have expected bonding to be between pure *p*-orbitals. Instead one can see that around Ge atoms there is an increase in CDD along the cube diagonals with the isosurfaces very similar to those representing covalent bonds between Ge and Te atoms, which is a clear indication of strong *s*–*p* mixing corresponding to *sp*^3^-hybridization with one of the hybridized orbitals being non-bonding (lone-pair). Use of ELF isosurfaces for the same structures is shown in Fig. 4S, 5S. The ELF vizualization shows lone-pair isosurfaces similar to those in CDD but does not show covalent bonds and hence is less appropriate when one wants to discuss bond switching processes.

In this work we investigate the dynamics of the amorphisation process with a special accent on the evolution of chemical bonding between the atoms and provide the answer to the crucial *how*-question. Based on the results of DFT simulations, we demonstrate that the amorphisation-crystallisation processes take place via the formation of transient Ge-Ge-Te TCBs in the excited state, a process made possible by the presence of LP electrons, followed by the formation of valency alternation pairs (VAPs). (See METHODS for details). Our findings (i) show strong similarities between structural changes in chalcogenide glasses and phase-change alloys, (ii) provide an atomistic explanation to the long existing conundrum of how the experimentally observed ultra-fast crystallisation on a ns time-scale in device structures^38^ co-exists with the high activation energy of ca. 2.3 eV determined from measurements at lower temperatures^39^ (extrapolation of the experimental data on crystallization kinetics to operating temperatures suggests a crystallization speed a couple of orders lower than the experimentally observed), (iii) and offer new insights into the search for prospective memory materials.

## Results and Discussion

We start by considering the final amorphous structure *generated from the crystalline phase* using DFT simulations as described in^40^. The resulting structure is shown in the left panel of Fig. 3. Atoms participating in the formation of tetrahedral configurations are marked in a different color (Ge - bright green, Te - orange) than the rest of the atoms in the simulation cell. In the right panel, only the atoms that form a tetrahedral configuration are shown with other atoms being invisible. We note that the tetrahedrally coordinated Ge atoms are bonded to a pyramidally (three-fold) coordinated Ge atom (the latter configuration is also referred to as a defective octahedral site^27^,^28^). We shall use the 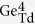
–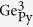 notation to describe this atomic configuration, where the superscripts correspond to the coordination numbers and the subscripts describe the geometry. The observation that tetrahedrally bonded Ge atoms have at least one Ge first-nearest neighbour (or Sb in case of Ge-Sb-Te), was also made for in-silico ‘melt-quenched’ amorphous GeTe (and Ge_2_Sb_2_Te_5_)^27^ and the stabilising role of Ge-Ge bonds on tetrahedral sites was stressed in^32^. It is informative to note that the Te atom located (almost) along the Ge-Ge bond becomes two-fold coordinated and the corresponding Ge-Te distance increases to ca. 3.1 Å, which is much longer than a typical Ge-Te covalent bond (2.61 Å). In other words, the homopolar Ge-Ge covalent bond is likely established at the expense of the original Ge-Te covalent bond. How are such configurations formed?

It should be noted that even though the bonding angles between the Ge and Te atoms are very close to 90°, the bonds are not between purely *p*-orbitals as often considered for simplicity, but *s-p* mixing occurs^28^, i.e. amorphous GeTe can be seen as a LP semiconductor with LP-electrons located on partially *sp*^3^-hybridised Ge orbitals^41^. The extent to which this orbital protrudes from the atomic core depends on the bond angles^42^. Local stresses during the relaxation process that increase the bond angle towards 109° concomitanly increase the extent to which the LP-electrons protrude.

The process of the formation of a tetrahedrally coordinated Ge site is illustrated in Fig. 4. In the upper panel, we show a schematic of the process. When a Ge atom (*A*) possessing an extended *s-p* mixed LP-orbital becomes aligned with a covalent Ge-Te bond between atoms *B* and *C* (left panel), a Ge-Ge-Te (*A*–*B*–*C*) TCB can be generated (center), with the subsequent formation of a Ge-Ge (*A*–*B*) bond, concomitant with the rupture of the Ge-Te arm of the TCB (right). The additional Ge-Ge bond is thus indeed created at the expense of the Ge-Te bond. As in the case of chalcogenide glasses, the key point is that the formation of the homopolar Ge-Ge bonds does not require precursory rupture of strong two-center covalent Ge-Te bonds. The subsequent destruction of the Ge-Te arm of a TCB actually serves to strengthen the remaining Ge-Ge bond and thus stabilises the amorphous phase. Schematically this process can be described as:


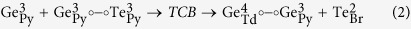


where the sign depicts covalent bonds, the superscripts describe the covalent coordination and the subscript *Br* refers to a bridging Te configuration; the subscripts *Py* and *Td* correspond to the pyramidal and tetrahedral configurations, respectively.

The proposed bond-switching model via the TCBs is substantiated by DFT simulations through the use of CDD isosurfaces. We remind the readers that CDD is the difference in electron density between the structure in question and non-interacting quasi-atoms. Consequently, the formation of a CDD cloud midway between two atoms is a signature of a covalent bond. Non-bonding lone-pair electrons located on both *p*- and *sp*^3^- orbitals are also clearly visualised using this approach. In the middle part of Fig. 4 we show the evolution of the CDD isosurfaces during the *in-silico* amorphisation process. Note that only the atoms that form two neighbouring GeTe ‘cubes’ in the starting crystalline model and the corresponding CDD isosurfaces are shown (other atoms in the simulation cell have been made invisible for the clarity of presentation). As the long-range order just starts to collapse and the average structure is still crystal-like, the CDD clouds are concentrated in-between Ge and Te atoms as in the crystalline phase. Additionally, one can also see a CDD cloud at the Ge atom (marked *A* in the Figure) directed along the diagonal of the GeTe ‘cube’, corresponding to a LP localised on a *sp*^3^-hybridised orbital. As the relaxation proceeds and two Ge atoms approach each other (central panel) such that the interatomic Ge-Ge and Ge-Te distances become nearly equal, a three-center bond is clearly generated as evidenced by CDD clouds below and above the central Ge atom (marked *B*). Finally, the Te atom (marked *C*) moves away (to a distance of 3.08 Å) and the CDD cloud characteristic of the original Ge-Te covalent bond disappears. The Te atom becomes two-fold coordinated, the preferred coordination for chalcogen species. Concurrently, the two-lobe CDD associated with the Te LP *p*-orbital becomes visible (right). The three panels in the lower row are zoomed regions in the vicinity of the atoms that participate in the formation of TCBs. The interatomic distances corresponding to the three configurations involved are also shown. These results provide unambiguous evidence of the formation of a transient state with three-center bonds during the amorphisation process.

It is interesting to note that the total number of covalent bonds does not change during this process and since the bond energies of the Ge-Ge and Ge-Te are rather similar, the total energy of the system is essentially unchanged, indicating the comparable stabilities of the two phases. The fact that the coordination of the Ge atom increases (three-fold to four-fold) while that of the Te atom decreases (three-fold to two-fold) demonstrates that a VAP has been created. This observation is in good agreement with earlier simulations where the *average* coordination numbers of Ge and Te increase and decrease, respectively, with respect to the three-fold covalent coordination in the crystalline phase^27^,^28^, and also with experimental extended x-ray absorption fine structure (EXAFS) results that found the average coordination numbers of Ge and Te in the amorphous phase to be *N*_*Ge*_ ≈ 4 and *N*_*Te*_ ≈ 2, respectively^34^,^33^. This compares well with the experimental observation of dynamical bonds in Se^15^ and further demonstrates the remarkable similarity between chalcogenide glasses and phase-change alloys. It may also be worth noting that the dramatic change in bonding between the crystalline and amorphous phases manifests itself in Raman scattering. Mode softening in the crystalline phase, observed for both chalcogenide glasses^43^ and phase-change alloys^26^ further underscores the similarity between them.

It is significant that in chalcogenide glasses (Se being the simplest example), atoms in the ground state possess the coordination number of two as required by the element’s valency and are neutral, while the atoms that form a VAP have ‘defective’ coordination numbers and are charged. In contrast, in the ground (crystalline) phase of GeTe, the 3 (+3) coordination is rather unusual for both Ge and Te, while the atoms that form a VAP possess the coordination numbers required by the 8–N rule and are *neutral*. This may be the underlying reason why as many as up to 50% of (Ge) atoms can acquire tetrahedral bonding geometry in melt-quenched phase-change alloys^31^, while the concentration of native VAP defects in chalcogenide glasses is typically on the order of 10^18^ cm^−3^. VAPs thus have a deterministic effect on the properties of the amorphous phase of the phase-change alloys and, in particular, similar to chalcogenide glasses they pin the Fermi level in the middle of the gap, as originally proposed in^44^. While, different from chalcogenide glasses, where the electronic state associated with VAPs are located inside the gap, in phase-change alloys, the valence alternation pairs were found to give rise mainly to states resonant with the valence and conduction bands, rather than in the band gap^45^; this behavior is consistent with the generalised negative-U model proposed by Anderson^46^.

The very fast crystallisation process of phase-change alloys is also determined by the TCB. Crystallisation of a tetrahedrally bonded semiconductor such as GaAs (Fig. 5, upper panel (a)) requires the rupture of strong covalent Ga-Ga and As-As bonds (panel (b)) *as a prerequisite* to the formation of bonds required by the material’s stoichiometry (panel (c)), followed by establishment of long-range crystalline order. This process is energetically costly (ca. 2 eV) and proceeds slowly. At the same time, in a phase-change alloy (lower panel), small atomic motion suffices for the formation of a TCB *without the need to provide any additional energy*. The subsequent rupture of the weaker arm of the TCBs during the relaxation process requires comparatively little energy and can be very fast, which accounts for the low activation energy at operating temperatures, recently observed using ultra-fast differential scanning calorimetry (DCS) measurements and attributed to the liquid phase being fragile^47^.

Subsequent ordering of the pyramidal configurations completes the crystallisation process. The different nature of bond switching during the crystallisation process in classic semiconductors and phase-change alloys is also reflected in rather different heats of crystallization: 0.95 kJ/mol for Ge_2_Sb_2_Te_5_^39^ vs. 11.9 kJ/mol for Si^48^.

It is crucial that the TCBs can only be established when atoms are sufficiently mobile in a thermally or electronically excited state in order to form the aligned configurations. At lower temperatures, bond switching requires rupture of the conventional two-center bonds, which is an energy costly process determining the higher activation energy. The formation of TCBs at elevated temperatures provides a natural explanation for the experimentally observed decrease in the crystallisation activation energy at elevated temperatures. It is interesting to note that while within the model of a fragile liquid the activation energy changes gradually^47^, some analyses suggested^49^ that a two-Arrhenius slope model is a better description of the behaviour of viscosity as a function of temperature, in perfect agreement with the proposed TCB concept.

The valency alternation of chalcogenide alloys, made possible thanks to the presence of LP-electrons^10^ and common to both chalcogenide glasses and phase-change alloys, is thus key to low-energy structural transformations. Not surprisingly, all known *functional* PCM alloys contain chalcogen atoms. What is crucial for fast switching is the ability of atoms to form transient three center bonds, the resulting VAP formation determines the high stability of the two phases. If one can artificially make a PCM material with Ge atoms located close to each other so that the Ge^Td^–Ge^Py^ configurations can be generated without significant atomic diffusion, one can expect further lowering of switching energy, which, indeed, has been observed in so called interfacial phase-change materials (iPCM)^50^. It may be worth noting that the transition between the SET and RESET states in iPCM does not involve melting. The easy formation of Ge_Td_–Ge_Py_ configurations is demonstrated in Fig. 6S (Suppl. Info), which shows a snapshot of molecular dynamics in an iPCM structure.

It is also important to control the concentration of the non-bonding LP-electrons *ψ*. As mentioned above, good glass formers are characterised by the value of *ψ* in a 0.5 to 0.66 range. Such materials do not crystallise easily and hence are not suitable for memory applications. On the other hand, the absence of LP electrons (e.g. in Si) results in its explosive crystallisation^51^, which is also unacceptable for a memory material that requires high stability of the amorphous phase. The intermediate value of *ψ*_*GeTe*_ = 0.4^14^ makes GeTe-based alloys ideal materials, which on the one hand are stable in the amorphous phase and at the same time exhibit a fast crystallisation speed. Varying the *ψ* value through doping appears to be a promising way to control the thermal stability of the amorphous phase alongside with the crystallisation speed where one can tune the properties of a phase-change material to particular applications.

Finally, our results suggest that one may reversibly control the direction of the process, i.e. which arm of the TCB breaks (Eq. 3), by creating conditions that favour one or the other structure, for example through use of coherent phonon excitation.


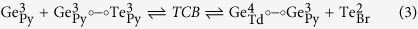


In view of the atomistic process discussed in this work, one can easily understand that crucial properties of phase-change memory alloys needed for industrial applications such as the low switching energies and the very high cyclability, are both determined by the formation of the dynamic three-center bonds that require little input energy and are soft and hence can accommodate stress generated at crystalline-amorphous boundaries. At the same time, the unchanged number of covalent (two-center) bonds in the two phases accounts for the fact that the stability of the amorphous phase is comparable to the stability of the crystalline phase.

Important questions are whether and how the dynamic TCBs can be studied experimentally. The time scale of the bond switching process would make such measurements very challenging but recent experimental progress using structure sensitive ultrafast experiments, e.g. using free-electron lasers, shows promise. Indeed, it was recently found that the photo-induced change in the diffraction peak intensity under intense sub-picosecond excitation of GeTe in the pre-amorphisation regime could not be explained by considering only heating effects and assuming an unchanged average structure^52^. We believe, however, that one of the best methods to provide spectroscopic signatures for the TCBs would be femtosecond time-resolved EXAFS measurements, because EXAFS is an element selective local probe that takes a snapshot of the structure on the 10^−15^ sec. time scale^53^, that is similar to the bond-switching time. In addition, TCBs, being very soft, should manifest themselves in increased polarizability, which might be seen in ultra-fast time resolved optical measurements. Such experiments are underway.

## Conslusions

In conclusion, by taking into consideration the chemical aspects of the dynamics of the phase-change process, we have demonstrated that the structural transformations taking place in both classes of chalcogenides under thermal or electronic excitation have fundamentally the same mechanism: bond switching via the formation of a transient phase with LP-mediated transient TCBs with the resulting formation of VAPs. The TCBs allow bond switching *without rupture of the existing strong covalent bonds as a prerequisite for the establishment of new bonds*, which makes the transformation process energy efficient. At the same time, in order for the transient bonds to form, the system has to be in a thermally (or electronically) excited state so that atomic positions can easily re-adjust. At lower temperature, both structures are very stable, which ensures the high stability of phase-change memory devices. Controlling the ability of a material to form TCBs and VAPs through changing the concentration of *p*-orbital LP electrons or the degree of extension of *sp*^3^-orbital LP electrons opens the possibility of tuning the material’s properties to particular applications. Additionally, our results suggest that one can control the direction of the process by creating conditions that favour the rupture of a particular arm of the TCBs.

One of the authors (AK) would like to acknowledge numerous discussions with S.R. Ovshinsky, who always pointed to the very important function of LP-electrons in memory materials (see e.g.^54^), but their role has largely been neglected. It is also AK’s great pleasure to acknowledge a discussion of this work with H. Fritzsche.

## Methods

**Figure 2:**
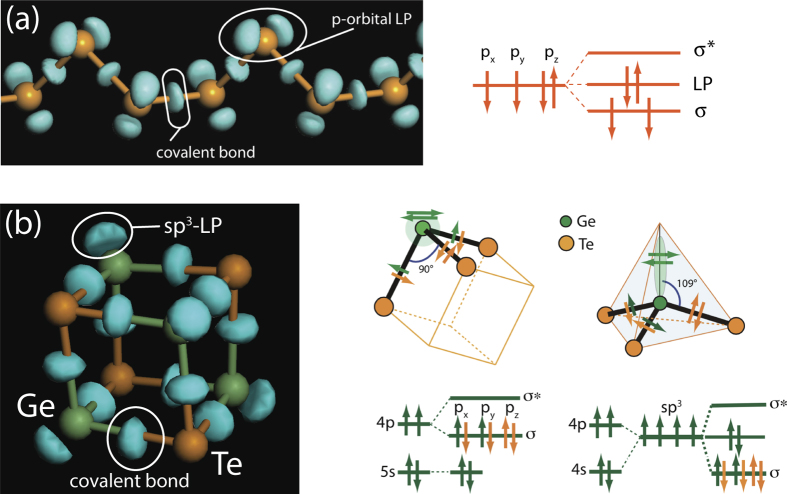
Charge density difference (CDD) isosurfaces for a Se chain (**a**) and a Ge_4_Te_4_ cube (**b**) alongside the corresponding energy diagrams showing the distributions of valence electrons. For the case of Ge-Te bonding cartoons showing pure *p*-bonding and *sp*^3^-hybridized bonding are also shown with electrons shown as arrows, where the color corresponds to the origin of the electron. Note that the use of CDD helps visualize *both* covalent bonds and lone-pair electrons. The clear presence of CDD clouds subtended at the Ge atoms along the cube diagonals is evidence of *sp*^3^-hybridisation despite the presence of near 90° bonding angles.

**Figure 3:**
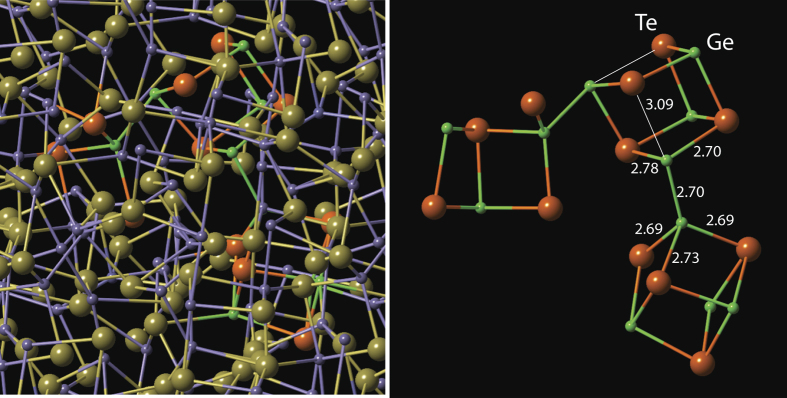
The structure (left) of the amorphous phase of GeTe obtained from the crystalline phase through the destruction of the longer bonds using DFT simulations. The majority of Ge atoms are shown in violet, and the majority of the Te atoms are shown in olive color. To make the atoms involved in the formation of tetrahedrally coordinated species stand out, the Ge atoms participating in tetrahedral configurations have been rendered in bright green while the Te atoms have been represented as orange spheres. The right panel shows only those atoms that are involved in tetrahedral Ge configurations, other atoms have been made invisible. One can see that the Ge-Ge bonds exist between a tetrahedrally coordinated Ge atom and a pyramidally bonded Ge atom. While all bonds for the tetrahedrally coordinated Ge atom and the three shorter bonds for the pyramidally coordinated Ge atoms are near-equal (the numbers are in Å), the fourth interatomic distance for the pyramidally (defective octahedral) coordinated Ge atom is significantly longer.

**Figure 4:**
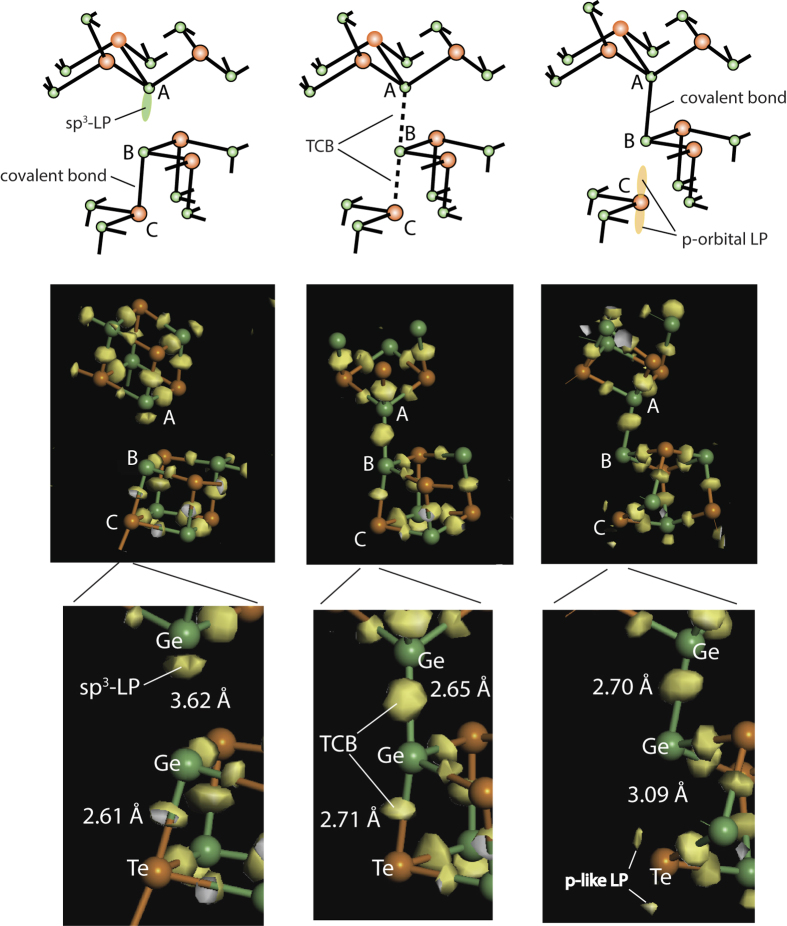
Upper row: schematic of the formation of a tetrahedral Ge configuration. As in Fig. 2, Ge atoms are shown in green and Te atoms are shown in orange. When a Ge atom with a protruding LP-orbital (marked *A*) comes close to another Ge atom (*B*) and is aligned with the neighbouring Ge-Te bond (between atoms *B*–*C*) (left panel), a three-center *A*–*B*–*C* bond is established (middle), whose subsequent rupture at the opposite arm results in the formation of a Ge_Td_–Ge_Py_ configuration (between atoms *A*–*B*), leaving behind a two-fold coordinated Te atom (*C*) (right). Middle row: evolution of CDD clouds during the in-silico amorphisation process using DFT simulations substantiating the schematic shown in the upper panel (see text for details). CDD clouds corresponding to the LP-electrons of an *sp*^3^ hybridised Ge orbital and a Te lone-pair p-orbital can be seen in the left and right panels respectively in addition to increased CDD midway between Ge and Te atoms that are signatures of covalent bonds. The presence of CDD clouds on both sides of the Ge atom (marked B in the figure) in the central panel is evidence of the formation of a transient three-center Ge-Ge-Te bond. Lower row: zooms into vicinities of the atoms that participate in the formation of TCB.

**Figure 5:**
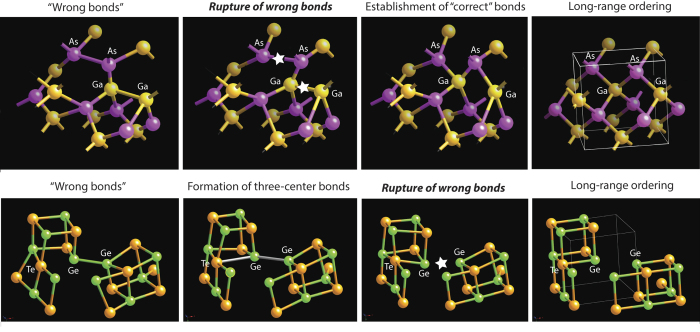
Comparison of the crystallisation processes of a tetrahedrally bonded semiconductor (GaAs, upper panel) and a phase-change alloy (GeTe, lower panel). Process evolution is shown from left to right. While in a classic covalent semiconductor, rupture of the ‘wrong’ bonds necessarily present in the amorphous phase is a *prerequisite* for the establishment of the bonds required by stoichiometry, in a phase-change alloy, the ‘wrong’ bonds break *after* the establishment of stoichiometry-required bonds (as one arm of a TCB). Because of the softness of TCBs, the latter process requires significantly less energy and accounts for the very fast crystallisation speed of phase-change alloys.

To analyse charge localisation and bonding between the atoms, various approaches can be used, such as the electron localisation function^36^, maximally localized Wannier functions^55^, the inverse participation ratio^56^, and charge-density difference (CDD)^37^,^57^ to name a few of the most often used techniques. In this work we use the CDD approach because it can visualise *both* the covalent bonds and LP electrons (Cf. Figs. 2 and 4S, 5S).

DFT calculations were carried out on a 64-atom cell using the plane-wave code CASTEP^58^. Ultrasoft pseudopotentials were used. The Ge and Te pseudopotential included the Ge 4s2 4p2 and the Te 5s2 5p4, as valence electrons, respectively. The exchange term was evaluated using the local density approximation from the numerical results of Ceperley and Alder as parametrized by Perdew and Zunger. The CDD was calculated with a plane-wave cutoff of 220 eV and a 2 × 2 × 2 Monkhorst-Pack grid.

## Additional Information

**How to cite this article**: Kolobov, A.V. *et al.* Understanding Phase-Change Memory Alloys from a Chemical Perspective. *Sci. Rep.* **5**, 13698; doi: 10.1038/srep13698 (2015).

## Supplementary Material

Supplementary Information

## Figures and Tables

**Figure 1:**
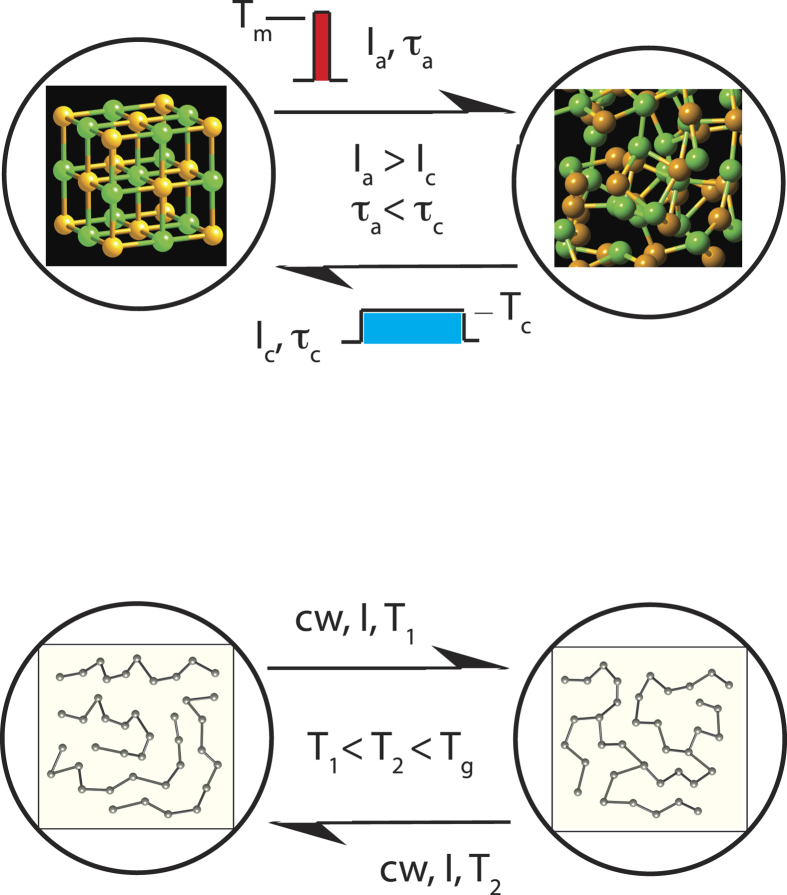
Comparison of crystallisation-amorphisation in a phase-change alloy (upper panel) and reversible photostructural change in a chalcogenide glass (lower panel). In the former case, the transition is induced by laser or current pulses of different intensity and duration that heat the material to different temperatures (above the melting point, *T*_*m*_, and the crystallisation temperature, *T*_*c*_). In the latter case, the structural change is induced by cw-light of low intensity (<*T*_*g*_); the process can be partially reversed by changing the temperature or the wavelength of the inducing light or completely reversed by annealing just under the glass-transition temperature *T*_*g*_. The material is always in the amorphous state.
