# Supplementary material for: Understanding Phase-Change Memory Alloys from a Chemical Perspective
Source: Sci Rep. 2015 Sep 1;5:13698. doi: 10.1038/srep13698 (PMC4555180; doi:10.1038/srep13698)
Supplement: Supplementary Information [file srep13698-s1.pdf]

SUPPLEMENTARY INFORMATION for "Understanding Phase-Change Memory Alloys from a Chemical Perspective"

A.V. Kolobov, P. Fons, J. Tominaga

Nanoelectronics Research Institute, National Institute of Advanced Industrial Science and Technology (AIST), 1-1-1 Higashi, Tsukuba 305-8562, Japan

(Dated: May 11, 2015)

LP ELECTRONS AND BOND SWITCHING

As mentioned in the main text, Dembovsky proposed in the early 1980s that LP electrons play a crucial role in glass formation of chalcogenides [1, 2]. It was noted that good glass-formers usually possess a high concentration of lone-pair electrons. This very specific chemical feature of chacogenides is usually ignored when the glass-transition in chalcogenides is discussed (see e.g. [3]). The glass transition temperature, i.e. the temperature above which the material flows (which corresponds to the viscosity value of 10^{12} Pa·s), is very low for selenium ($T_g \approx 30$ °C). What determines its ability to flow when the supplied thermal energy is not sufficient to break the Se-Se covalent bonds (172 kJ/mol [1])? For comparison, for tetrahedrally bonded silicon T_g=1000 K [4], while the Si-Si bond energy is just slightly larger (ca. 220 kJ/mol). Moreover, there is experimental evidence that the average coordination number of selenium slightly *increases* in the liquid phase [1], despite the shortening of chains in the melt accompanied by an increased concentration of singly coordinated atoms at chain ends, suggesting that the average coordination should rather decrease. Dembovsky proposed that the high viscosity of glassy melts alongside with high atomic mobility is determined by the formation of TCBs [1]. When a selenium chain end in the melt approaches a neighbouring chain such that three atoms become aligned in the way shown in Fig. 3S (left panel), LP electrons of the left (orange) Se atom resonate with the covalent bond between the middle (blue) and right (magenta) atoms and a TCB can be created. Once the TCB is established (middle panel), the left and right atoms become identical from the point of view of the central atom, namely, there are two electrons on each side of the central atom located on overlapped p-orbitals aligned along the three-atom axis. TCBs are soft and, provided the amplitude of thermal vibrations is large enough at the glass-transition temperature, one of its arms breaks, leaving behind a stronger two-center covalent bond and generating a new singly coordinated chain end Se atom (right panel). The net result is significant diffusion of the chain-end atom *without actual atomic displacement*.

In a similar way, when three normally (two-fold) coordinated Se atoms in neighbouring chains are properly aligned as shown in Fig. 4S, an interchain bond can be formed (as an arm of a TCB) with the subsequent formation of a so-called valence alternation pair (VAP), which consists of a positively charged three fold-coordinated (C_3^+) and a negatively charged singly coordinated (C_1^-) Se atom [5–9], whereas the majority of atoms are two-fold

coordinated and neutral (C_2^0). The $C_3^+C_1^-$ atoms that form a VAP can either recombine or, alternatively, the singly coordinated atom can 'diffuse' away resulting in a frozen-in concentration of VAPs on the order of 10^{17} – 10^{18}cm^{-3} [10].

COVALENT BOND AND LP VISUALISATION

Covalent bonds and lone-pair (LP) electrons can be visualised using different approaches such as the charge density difference (CDD), the electron localisation function (ELF), and the maximally localised Wanner orbitals. We have chosen here the CDD approach because it can show both the covalent bonds *and* LP electrons in the same image. This is illustrated below where we compare CDD and ELF in Fig.1S for the case of selenium for LP p-orbitals and in Fig. 2S for the case where LP electrons are located on an sp^3-hybridised orbital.

INTERFACIAL PCM

Spatial separation of GeTe and Sb_2Te_3 layers into so-called interfacial phase-change memory (iPCM) structure results in a significant decrease of energy consumption [11]. The lowest energy iPCM structure has been shown [12] to possess the stacking sequence as shown in Fig. 5S (left panel), where Ge atoms are shown in green, Te atoms in orange and Sb atoms in magenta. In this structure, Ge atoms form adjacent atomic planes and the establishment of Ge-Ge bonds can proceed more easily than in the alloy material of the same average composition. Molecular dynamics simulations demonstrated that at 700 K the Ge^{Td}-Ge^{Py} configuration is established on a ps-timescale (Fig. 5S, right panel).

[1] S. A. Dembovsky, Mat. Res. Bull. **16**, 1331 (1981).

[2] S. A. Dembovsky and E. A. Chechetkina, *Glass Formation (in Russian)* (Nauka, 1990).

[3] J. C. Mauro and R. J. Loucks, Phys. Rev. B **76**, 174202 (2007).

[4] J. Robertson, Can. J. Phys. **92**, 553 (2013).

[5] M. Kastner, D. Adler, and H. Fritzsche, Phys. Rev. Lett. **37**, 1504 (1976).

[6] D. Vanderbilt and J. Joannopoulos, Phys. Rev. B **22**, 2927 (1980).

[7] D. Vanderbilt and J. Joannopoulos, Phys. Rev. Lett. **42**, 1012 (1979).

[8] D. Vanderbilt and J. Joannopoulos, Physical Review B **23**, 2596 (1981).

[9] X. Zhang and D. Drabold, J. Non-Cryst. Solids **241**, 195 (1998).

[10] N. F. Mott and E. A. Davis, *Electronic Processes in Non-Crystalline Materials* (Clarendon Press Oxford, 1979), 2nd ed.

[11] R. E. Simpson, P. Fons, A. V. Kolobov, T. Fukaya, M. Krbal, T. Yagi, and J. Tominaga, Nature Nanotech. **6**, 501 (2011).

[12] J. Tominaga, A. V. Kolobov, P. Fons, T. Nakano, and S. Murakami, Adv. Mat. Interf. p. 1300027 (2014).

[13] M. Xu, Y. Cheng, H. Sheng, and E. Ma, Phys. Rev. Lett. **103**, 195502 (2009).

[14] M. Krbal, A. V. Kolobov, P. Fons, J. Tominaga, S. R. Elliott, J. Hegedus, and T. Uruga, Phys. Rev. B **83**, 054203 (2011).

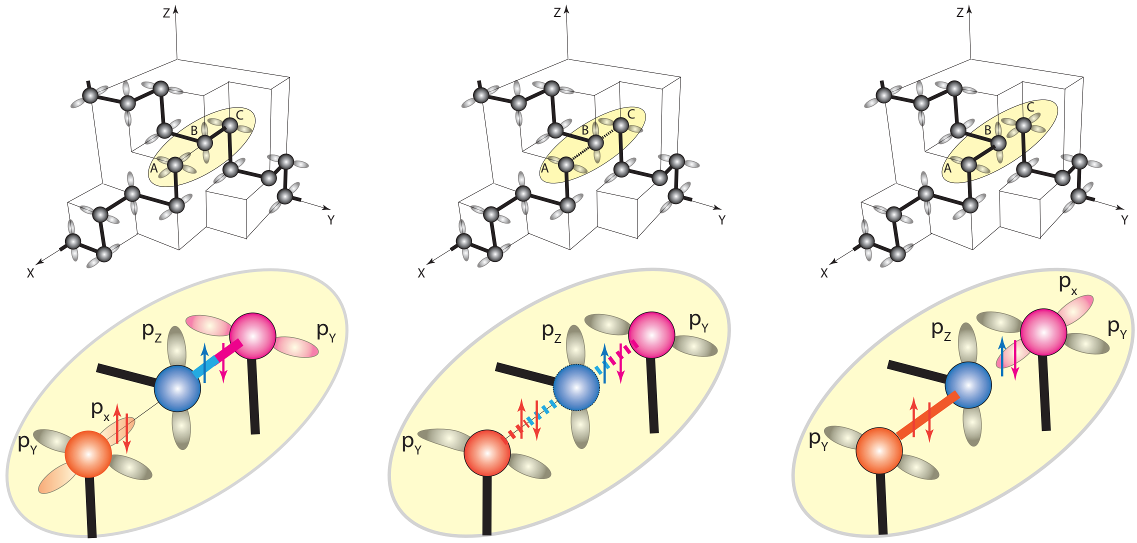

FIG. 1: Schematics of viscous flow in selenium melt enabled through the formation of TCBs (see text for details). In the lower panel Se atoms directly participating in the formation of a TCB are shown as differently coloured spheres. Conventional two-center covalent bonds are shown as solid lines (the colour corresponds to the atom providing electron(s) for bonding). Valence p-electrons participating in bonding are also marked in different colours corresponding to their source (while, of course, they are completely indistinguishable). The 8-shaped clouds depict LP p-orbitals.

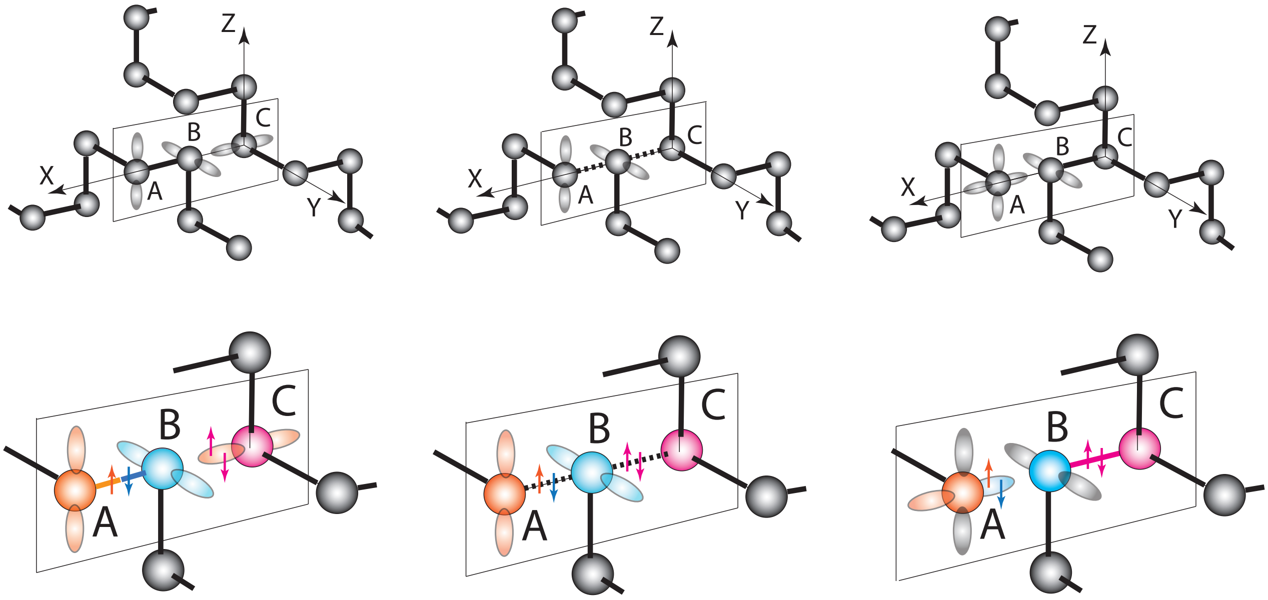

FIG. 2: Schematic illustration of the formation of VAPs in a selenium melt or during photostructral change (see text for details) via the formation of a TCB. As in Fig. 2, the participating atoms and valence electrons are marked in three different colours.

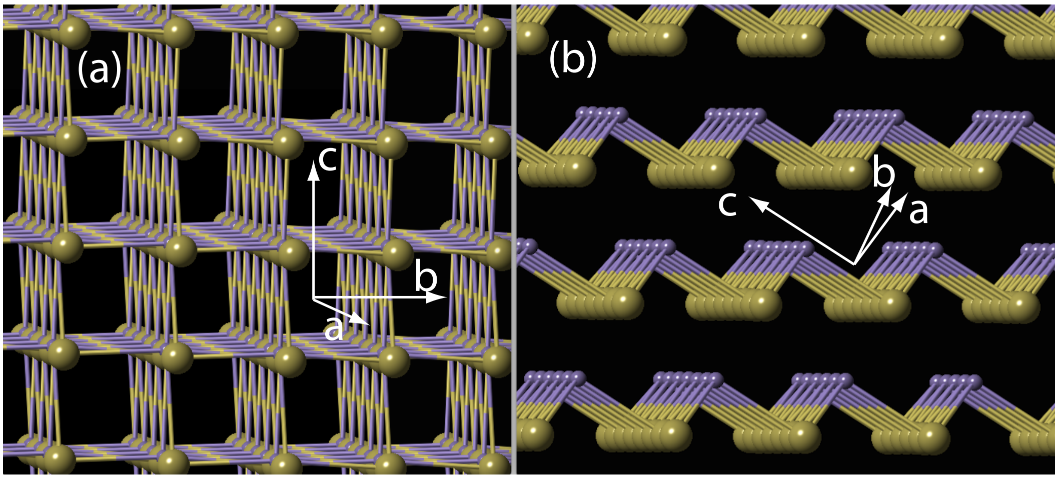

FIG. 3: Ball-and-stick representations of the rhombohedral GeTe structure (Ge atoms - violet, Te atoms - olive) with bond length cut-off distances of 3.1 Å (left) and 3.2 Å (right). Pseudo-cubic a,b, and c crystal axes are also shown. Different crystal orientations are shown for better visualization. One can see that in one case all atoms appear to be six-fold coordinated within a three-dimensional structure, while in the other case the same atoms are three-fold coordinated and the structure is layered two-dimensional. Such strikingly different conclusions arising from just 0.1 Å difference in the bond length cut-off clearly demonstrate insufficiency of the ball-and-stick approach.

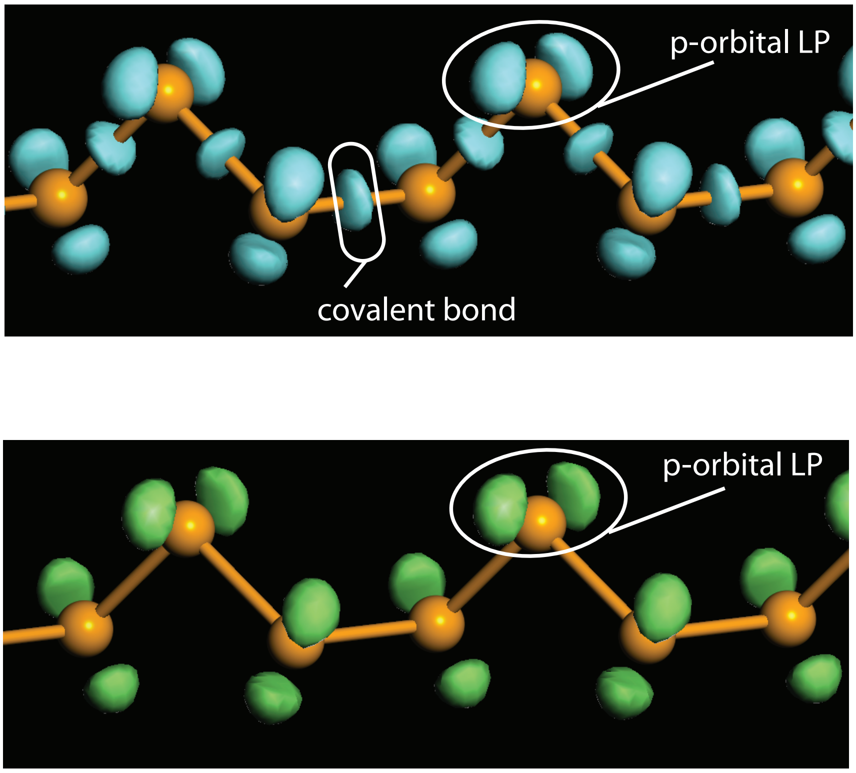

FIG. 4: An isolated selenium chain (orange colour) and the associated CDD (upper panel) and ELF (lower panel) isosurfaces simulated using DFT calculations. One can see that the CDD clouds appear for both covalent bonds and LP electrons, while the ELF isosurfaces only depict LP electrons.

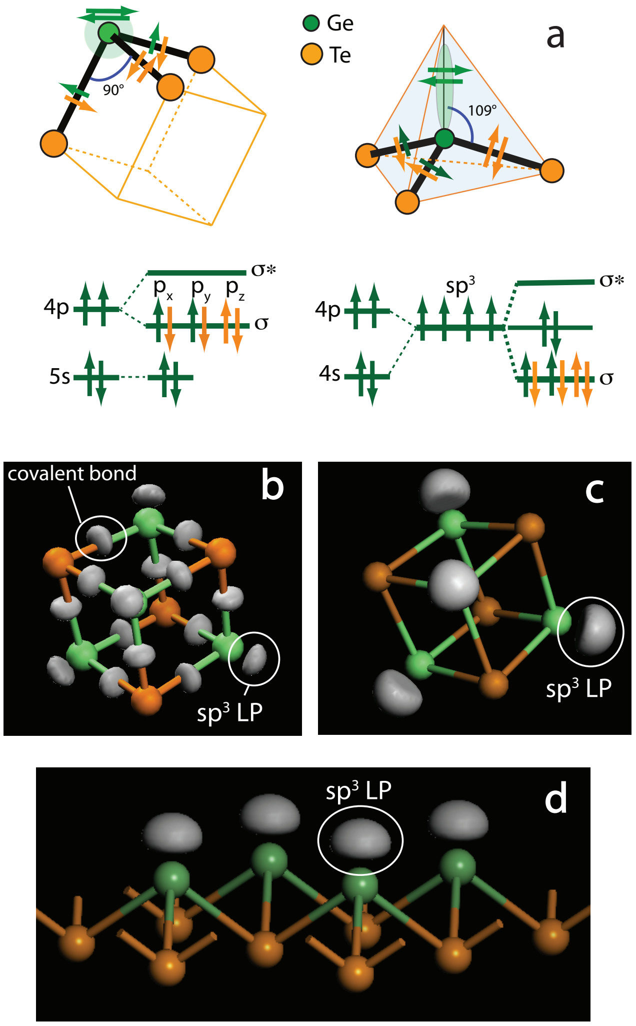

FIG. 5: (a) Schematic of the ideal p- (left) and sp^3- (right) Ge-Te_3 bonding configurations and the corresponding electronic configurations of Ge atoms. Ge atoms - green, Te atoms - orange. Valence electrons are shown as arrows with matching colour. The paired orange arrows indicate dative bonds (see [13, 14] for details) where both electrons are provided by Te species. While in the former structure, the LP electrons reside on a deeper lying s-orbital, in the latter they are located on the outer sp^3-hybridised orbital. (b) and (c) show DFT-simulated CDD and ELF isosurfaces, respectively, for an imaginary cubic Ge_4Te_4 molecule; despite the bonding angle being very close to 90°, the CDD and ELF isosurfaces clearly indicate a significant degree of s-p mixing. (d) shows ELF for a single GeTe layer, that is a building block of the rhombohedral phase of GeTe, also demonstrating the presence of LP electrons associated with s-p mixing

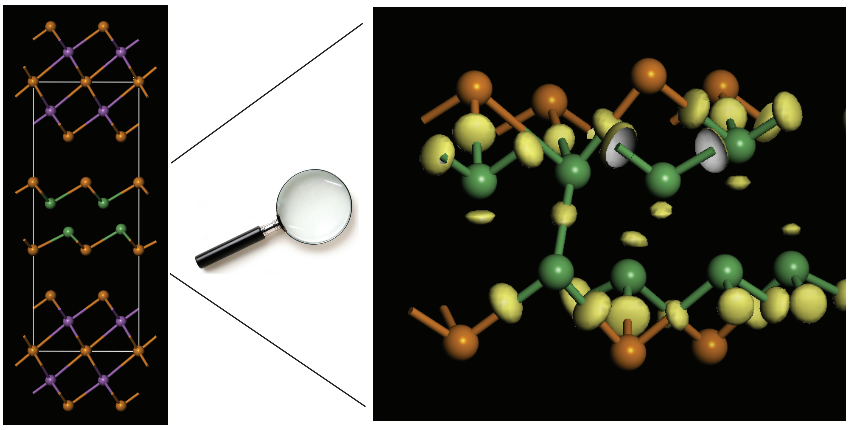

FIG. 6: The stacking sequence of the energetically most stable GeTe-Sb_2Te_3 structure fabricated at 500 K (left) and CDD isosurfaces within the GeTe block after 1 ps of annealing at 600 K (right). Ge - green, Sb - magenta, Te - orange. The formation of a Ge^{Td}-Ge^{Py} configuration is evidenced by an appearance of a CDD cloud midway between the two Ge atoms, in addition to the CDD clouds located between the Ge-and Te atoms and associated with Ge-Te covalent bonds. Other Ge atoms possess CDD clouds indicative of sp^3-hybridised LP-orbitals (in addition to those due to covalent bonds with Te species).
